# The Clinical Role of SIRT-3 in the Acute Rejection Process of Kidney Transplantation and Its Effects on Graft Outcomes: Evaluation of Biomarker Potential

**DOI:** 10.3390/medicina61030457

**Published:** 2025-03-06

**Authors:** Necip Altundaş, Eda Balkan, Murat Kizilkaya, Murat Altunok, Elif Demirci, Nurhak Aksungur, Salih Kara, Gürkan Öztürk, Abdullah Uyanik

**Affiliations:** 1 Department of General Surgery, Atatürk University, 25240 Erzurum, Turkeynurhak.aksungur@atauni.edu.tr (N.A.); skara@hotmail.com (S.K.); gurkanoztrk@yahoo.com (G.Ö.); 2 Department of Medical Biology, Atatürk University, 25240 Erzurum, Turkey; muratkizilkaya25@hotmail.com; 3 Department of İnternal Medicine, Atatürk University, 25240 Erzurum, Turkey; mrtalt@hotmail.com (M.A.); auyanik@atauni.edu.tr (A.U.); 4 Department of Pathology, Atatürk University, 25240 Erzurum, Turkey; elifpat@gmail.com

**Keywords:** kidney rejection, sirtuin, ELISA, mRNA

## Abstract

*Background and Objectives*: The aim of this study was to investigate the changes in the SIRT family, the effects of sirtuins on kidney graft function, and their potential as biomarkers in patients who develop rejection after kidney transplantation. *Materials and Methods*: Blood samples were collected from 45 kidney transplant patients before and after rejection. Some of these patients experienced T-cell-mediated early rejection (TCMR), while others presented antibody-mediated late rejection (ABMR). The mRNA expression levels of SIRT-1, SIRT-3, and SIRT-7 were measured via real-time PCR, while the protein levels of SIRT-1, SIRT-2, SIRT-3, SIRT-5, and SIRT-7 were assessed using ELISA. Patients were grouped based on rejection type and histological characteristics. Statistical analyses were performed using SPSS software (V23). *Results*: The mean age of the patient group was 42.22, while the control group had a mean age of 35.23 (*p* = 0.002). SIRT-1, SIRT-3, and SIRT-7 levels were significantly higher in patients with rejection (*p* < 0.001). In patients with late-stage rejection, SIRT-3 was found to be associated with interstitial fibrosis and C4d accumulation. SIRT-7 levels showed a weak correlation with potassium levels (*p* = 0.014). *Conclusions*: Our findings demonstrate significant changes in the SIRT family during both early- and late-stage rejection processes. Particularly, the role of SIRT-3 in the late stage is highlighted, suggesting the potential use of this gene as a biomarker for managing rejection processes. These findings could provide valuable insights for developing treatment strategies in organ transplantation.

## 1. Introduction

One of the best ways to treat end-stage renal disease is kidney transplantation, which can increase survival and enhance quality of life. However, graft health can be adversely affected by acute and chronic rejection mechanisms that arise after transplantation, which can result in organ loss. Improving transplantation success and guaranteeing long-term graft life greatly depend on early rejection identification and a knowledge of its processes. The accuracy of current diagnostic techniques is low, and in-depth molecular-level research is needed to comprehend the rejection processes [1].

The sirtuin family (SIRT1-7) is a group of proteins that function as NAD⁺-dependent deacetylases and ADP-ribosyltransferases. These proteins are involved in a wide range of biological processes, including energy metabolism, oxidative stress management, DNA repair, inflammation, and apoptosis [2]. For example, SIRT1 has been shown to regulate immune responses by suppressing inflammatory cytokine production, SIRT3 protects mitochondrial functions, and SIRT6 plays a role in the DNA damage response. SIRT2 is involved in cell cycle regulation and suppression of inflammatory responses, while SIRT5 supports mitochondrial functions and antioxidant defense. SIRT7 modulates cellular stress responses and plays a significant role in DNA repair and ribosomal biogenesis processes [3].

SIRT1 and SIRT3 have been found to mitigate sepsis-induced acute kidney injury by triggering mitochondrial autophagy [4,5]. SIRT7 can alleviate ferroptosis, fibrosis, and damage in the kidneys of hypertensive mice by supporting specific signaling pathways, while SIRT2 reduces damage in hypertensive nephropathy. These findings demonstrate that the sirtuin family plays a significant role in kidney diseases [6,7].

However, the molecular functions of the sirtuin family in kidney transplant rejection pathways are not yet fully understood. Specifically, it is still unclear how sirtuin expression levels are altered during rejection, and how these alterations impact graft function and relate to clinical outcomes.

The aim of this study was to investigate the relationship between the protein levels and gene expression of SIRT1, SIRT2, SIRT3, SIRT5, SIRT6, and SIRT7 in the rejection process following kidney transplantation and to explore the potential use of sirtuins as biomarkers in kidney transplant rejection processes and graft survival.

## 2. Material and Methods

### 2.1. Materials

#### 2.1.1. Study Scope and Design

This study was conducted in collaboration with the Departments of Organ Transplantation, Nephrology, Medical Biology, and Pathology at Atatürk University Faculty of Medicine. It had a retrospective and prospective design. The study included 45 patients diagnosed with rejection after kidney transplantation.

#### 2.1.2. Participants and Data Collection

Blood count and serum samples were collected from the patients in accordance with the regular post-transplant follow-up protocol. Demographic data (age, gender), type of transplantation (cadaveric or living donor), comorbidities, and clinical information (laboratory results, type and timing of rejection, and immunosuppressive treatment regimen) were recorded in detail.

In patients who developed cellular rejection under the immunosuppressive treatment regimen, initial treatment was initiated with corticosteroids. When necessary, calcineurin inhibitors such as tacrolimus or cyclosporine were added. High-dose steroid treatment was administered in cases where there was no response to steroid therapy.

In patients who developed humoral rejection, antibodies were removed through plasma exchange (plasmapheresis). Intravenous immunoglobulin (IVIG) therapy was administered, and rituximab was used when necessary. In our study, individualized treatment approaches were applied to patients in accordance with these treatment protocols.

The patients enrolled in this study included individuals who developed rejection at different time points after transplantation. A subset of patients experienced early T-cell-mediated rejection (TCMR), while others developed late antibody-mediated rejection (ABMR). Rejection was classified based on the time of onset as early (0–6 months) and late (>6 months) rejection. Early rejection includes acute cellular and humoral rejections that occur within the first 6 months of transplantation, while late rejection includes chronic rejection processes that arise after 6 months. This classification plays a significant role in the management and follow-up of patients based on the timing of the rejection process.

The patients included in the study were individuals diagnosed with early- and late-stage rejection. Participants were selected based on the clinical and pathological findings of rejection, and specific biological samples (e.g., kidney biopsy and blood samples) were obtained. Additionally, individuals with appropriate conditions for mRNA and ELISA analyses were selected. A group with similar general health status, age range, immunosuppressive treatment history, and graft function was formed to avoid potential confounding factors. The demographic characteristics, clinical, and laboratory data of the included individuals allowed for a more accurate examination of the effects of different rejection processes and sirtuin expressions.

Patients who did not meet the inclusion criteria were excluded from the study. These included individuals who did not have a rejection diagnosis, those who could not provide the necessary biological samples (e.g., kidney biopsy and blood samples), and patients for whom ELISA results or mRNA analysis results could not be obtained. Furthermore, patients who died due to graft loss, as well as those whose immunosuppressive treatment history could not be clearly determined and those with infections or other systemic inflammatory diseases, were not included in the study. These criteria were established to enhance the reliability of the data obtained and to avoid potential confounding factors. It was believed that the excluded patients did not provide data suitable for the study’s objectives and would have negatively impacted the accuracy and validity of the research.

### 2.2. Collection of Blood and Serum Samples

#### 2.2.1. Pre-Rejection Blood Samples

Blood and serum samples were collected at regular intervals before rejection occurred to predict the development of post-transplant rejection and evaluate molecular markers.

#### 2.2.2. Blood Samples at the Time of Rejection

Blood and serum samples were collected from patients who developed rejection at the time of rejection as well as during the early and late phases of rejection. The expression and levels of sirtuins were measured before the initiation of any drug therapy, and the obtained data were used as baseline information at the start of the treatment process. Blood and serum samples were collected according to the following standards:

- Blood samples were collected before the initiation of treatment (to ensure an accurate assessment of the status of sirtuins).
- Blood samples were collected in the morning during the fasting state at the time of rejection using EDTA-containing tubes.
- The samples were centrifuged at 3000 rpm for 10 min to obtain plasma.
- Plasma samples were stored at −80 °C.

Blood samples were analyzed twice for each patient to ensure the accuracy and reproducibility of the measurements.

### 2.3. Control Group

The control group consisted of 35 healthy individuals who were unrelated and had no systemic diseases. Blood and serum samples obtained from the control group were processed and stored using the same method.

### 2.4. Ethical Approval

This study was conducted in accordance with the Declaration of Helsinki and was approved by the Clinical Research Ethics Committee of Atatürk University Faculty of Medicine (Approval Code: B30.2 ATA-0.01.00/588, Approval Date: 25 October 2023). Written informed consent was obtained from all participants.

Throughout the study, SIRT-1, SIRT-3, and SIRT-6 gene expression levels were measured using real-time PCR, and protein levels were evaluated using ELISA for SIRT-1, SIRT-2, SIRT-3, SIRT-5, SIRT-6, and SIRT-7. The obtained data were compared with demographic, laboratory, and pathological data.

Statistical analyses were performed, and new findings regarding the molecular basis of rejection are presented.

### 2.5. Method

In our study, the clinical and laboratory parameters of kidney transplant patients experiencing rejection were recorded retrospectively and prospectively. In cases of suspected rejection, kidney biopsies were obtained from the patients, and relevant protocols were applied for pathological evaluation. Biopsy results were correlated with diagnoses of acute cellular and humoral rejection according to the Banff 2017 criteria.

#### 2.5.1. SIRT-1, SIRT-3, and SIRT-6 Expression Levels: Molecular Real-Time PCR Analysis

In our study, real-time PCR was used to determine the expression levels of the SIRT-1, SIRT-3, and SIRT-6 genes. A series of optimization procedures were applied to enhance the accuracy and reliability of the test. First, RNA isolation was performed using the QIAGEN (Hilden, Germany) RNeasy Plus Mini Kit, and cDNA synthesis was carried out with the QIAGEN QuantiTect Reverse Transcription Kit. The PCR amplification conditions were optimized to ensure the accurate replication of genetic material. For instance, the denaturation time, primer annealing temperatures, and the number of amplification cycles were adjusted to minimize error rates and maximize sensitivity. Additionally, both negative and positive control samples were included in each test to ensure the reliability of the results.

**RNA Isolation:**

RNA was isolated from the patient and control samples using the QIAGEN RNeasy Plus Mini Kit (Cat. No. 172045918). The isolation process was performed as follows: (a) 1 mL of blood collected from the patient and control samples was mixed with 5 mL of EL buffer (provided in the kit) and incubated on ice for 15 min. (b) After incubation, the tubes were centrifuged at 1900 rpm for 10 min, and the supernatant was carefully removed. (c) RLT buffer (600 µL) was added to the pellet, dissolving it completely. The mixture was then loaded onto lilac-colored spin columns (provided in the kit) in 700 µL aliquots and centrifuged at 13,000 rpm for 3 min. (d) Following centrifugation, 600 µL of 70% ethanol was added to the liquid portion remaining beneath the column, and the solution was transferred to a white spin column, which was then centrifuged at 10,000 rpm for 1 min. (e) The spin column was placed into a new tube, and 700 µL of RWI buffer was added, followed by centrifugation at 10,000 rpm for 1 min. (f) The spin column was transferred to another new tube, and 500 µL of RPE buffer was added before centrifugation at 14,000 rpm for 4 min. (g) The spin column was placed into a final new tube, and 40 µL of RNase-free water was added. A final centrifugation step was performed at 10,000 rpm for 2 min, completing the RNA extraction process. This kit efficiently extracts high-quality RNA from various tissue samples. The procedure involves carefully homogenizing tissue samples, followed by the addition of reagents to lyse cell membranes while preserving RNA integrity. The RNA was subsequently purified using a spin column and further treated with on-column DNase to remove any residual DNA contamination, ensuring the integrity of the genetic material.

**cDNA Synthesis:**

The RT^2^ First Strand Kit QIAGEN (Hilden, Germany)RNeasy was used to synthesize cDNA from the RNA obtained in the previous step. The kit protocol followed was as follows: (a) For each patient, 1 µL of GE buffer and 4 µL of RNA sample (containing 100 ng RNA per 1 mL) were mixed and incubated in a PCR device at 42 °C for 5 min. (b) After PCR incubation, the following components were added to the tubes: 2 µL of 5× BC3 buffer, 0.5 µL of Control P2, 1 µL of Reverse Transcriptase, and 1.5 µL of RNase-free water. The tubes were then placed in a PCR program set to 42 °C for 15 min followed by 95 °C for 5 min. (c) At the end of the reaction, cDNA was obtained. This kit utilizes oligo-dT primers and random hexamers to efficiently convert RNA into cDNA through reverse transcription. The reaction was carried out in a thermal cycler under the conditions recommended by the manufacturer, ensuring the complete conversion of RNA into cDNA.

**Real-Time PCR Amplification:**

In this step, the cDNA obtained in the previous phase was subjected to qPCR (quantitative PCR) using the QIAGEN QuantiTect SYBR Green PCR Master Mix (Cat. No. 208056). Each PCR reaction tube contained 6.5 µL of SYBR Green PCR Master Mix, 0.5 µL of Primer, and 5 µL of cDNA. The prepared reaction mixtures were then placed into a Qiagen Rotor-Gene qPCR machine for amplification.

**PCR Cycling Conditions:**

The PCR conditions were optimized to ensure accurate amplification and minimize primer-dimer formation:

- Initial denaturation: 95 °C for 15 min (to activate the polymerase).
- Amplification cycles (40 cycles):
  Denaturation: 95 °C for 15 s.
  Annealing (binding): 60 °C for 30 s.
  Extension: 72 °C for 30 s.
- Melting curve analysis was performed after amplification, ranging from 65 °C to 95 °C with 0.5 °C increments, holding for 5 s at each step.

**Data Analysis:**

The expression levels of the SIRT-1, SIRT-3, and SIRT-6 genes were determined by calculating ΔCT (delta cycle threshold) values. CT represents the number of cycles required for fluorescence to exceed a defined background threshold. ΔCT values were compared between the patient and control groups.

Gene expression levels were normalized to GAPDH, which served as an internal control to account for variations in RNA input and cDNA synthesis efficiency.

**Technical Replicates:**

Each sample was tested in duplicate to ensure consistency and reliability. The same PCR procedure was also applied to control group samples for comparative analysis.

**Kits and Primer Information Used in the Study:**

The following kits and primers were used in real-time PCR experiments:

- QIAGEN QuantiFast SYBR Green PCR Kit (Cat. No. 208056).
- QIAGEN RT^2^ First Strand PCR Kit (Cat. No. 77203139).

The primers used in the study were as follows:

- SIRT1: (ID No: QT01886675).
- SIRT3: (ID No: QT00091490).
- SIRT6: (ID No: QT00056812).

These primers were provided by QIAGEN (Hılden Germany) and were manufacturer-certified for specificity to each gene.

#### 2.5.2. SIRT1, SIRT2, SIRT3, SIRT5, SIRT6, and SIRT7 Expression Levels: ELISA-Based Analyte Assay Techniques

The Enzyme-Linked Immunosorbent Assay (ELISA) method was used to measure the protein levels of SIRT1, SIRT2, SIRT3, SIRT5, SIRT6, and SIRT7. The YLbiont Model ELISA Kit (Guangzhou, China) was selected for the ELISA assay. This assay enables the quantification of the specific protein content in each sample using specific antibodies for each protein.

At the beginning of the assay, serum and tissue samples (100 µL per well) were added to 96-well microtiter plates, which were incubated at room temperature for 30 min. This step was conducted to allow adsorption of the serum and tissue samples onto the surface of the plate. Following the initial incubation, wells were treated with SIRT1-, SIRT2-, SIRT3-, SIRT5-, SIRT6-, and SIRT7-specific antibodies. These antibodies bound to their target proteins, forming specific binding sites. After the antibody addition, the plates were incubated at 37 °C for 1 h. This process was carefully optimized to maximize the binding efficiency of the antibodies to the target proteins. At the end of the incubation period, the plates were washed three times with the buffer provided in the kit. This washing step was crucial for removing unbound antibodies and other components, thereby improving the accuracy of the measurements. After the washing step, horseradish peroxidase (HRP)-conjugated antibodies were added to each well. HRP conjugates interacted with the previously bound specific antibodies, initiating a chemical reaction in the final detection phase. Following the addition of HRP-conjugated antibodies, the plates were incubated at 37 °C for 30 min. The incubation time was carefully selected to ensure proper binding of HRP to the antibodies. At the end of the second incubation period, the plates were washed again to remove unbound HRP conjugates and other free components. Then, 100 µL of substrate solution was added to each well, and the reaction was allowed to proceed for 10 min. The substrate solution interacted with the HRP enzyme, generating a color change. This color change served as an indicator of protein levels in the sample. To stop the reaction, 100 µL of stop solution was added to each well. The stop solution halted the reaction and stabilized the color change. The resulting color intensity was measured using a microplate reader at 450 nm. The color intensity was directly proportional to the protein levels in the samples. These measurements were then compared to a standard curve provided in the kit to accurately determine the protein concentrations. To enhance accuracy and reliability, every step of the test was carefully monitored and optimized. Positive and negative control groups were included in each experiment to validate the results and ensure the reliability of the test. The optimal temperatures, incubation times, and washing steps were precisely determined, and stringent control was applied at each step, increasing the overall trustworthiness of the results. The test results were analyzed using the standard curve provided by the kit. At the end of each experiment, measurements and results were compared to determine the protein levels accurately.

##### Sirtuin Proteins and Catalog Numbers Used in This Study

The sirtuin proteins used in the study and their catalog numbers are as follows:

- SIRT1 (YLA3726HU).
- SIRT2 (YLA0774HU).
- SIRT3 (YLA1951HU).
- SIRT5 (YLA3220HU).
- SIRT6 (YLA1952HU).
- SIRT7 (YLA2986HU).

### 2.6. Statistical Methods

Statistical analysis was conducted using IBM SPSS V23. Normality was assessed using the Shapiro–Wilk and Kolmogorov–Smirnov tests. The Independent Samples *t*-test was used to compare normally distributed variables between groups, while the Mann–Whitney U-Test was applied for non-normally distributed variables. Categorical variables were compared using Yates’ Correction. The Wilcoxon test was employed to compare non-normally distributed variables measured at two different time points. The relationships between normally distributed variables were analyzed using Pearson’s Correlation Coefficient, whereas Spearman’s rho Correlation Coefficient was used for non-normally distributed variables. The Intraclass Correlation Coefficient (ICC) was applied to assess agreement between variables. To determine the cut-off values for disease identification, ROC analysis was performed, and the DeLong test was used to identify the most effective method for disease prediction. The results are presented as frequency (percentage) for categorical variables and as mean ± standard deviation or median (minimum–maximum) for quantitative variables. A *p*-value of <0.050 was considered statistically significant.

## 3. Results

The mean ages of the participants according to groups showed a statistically significant difference (*p* = 0.002). The mean age of the patient group was 42.22, while the mean age of the control group was 35.23 (Table 1).

**Figure 1:**
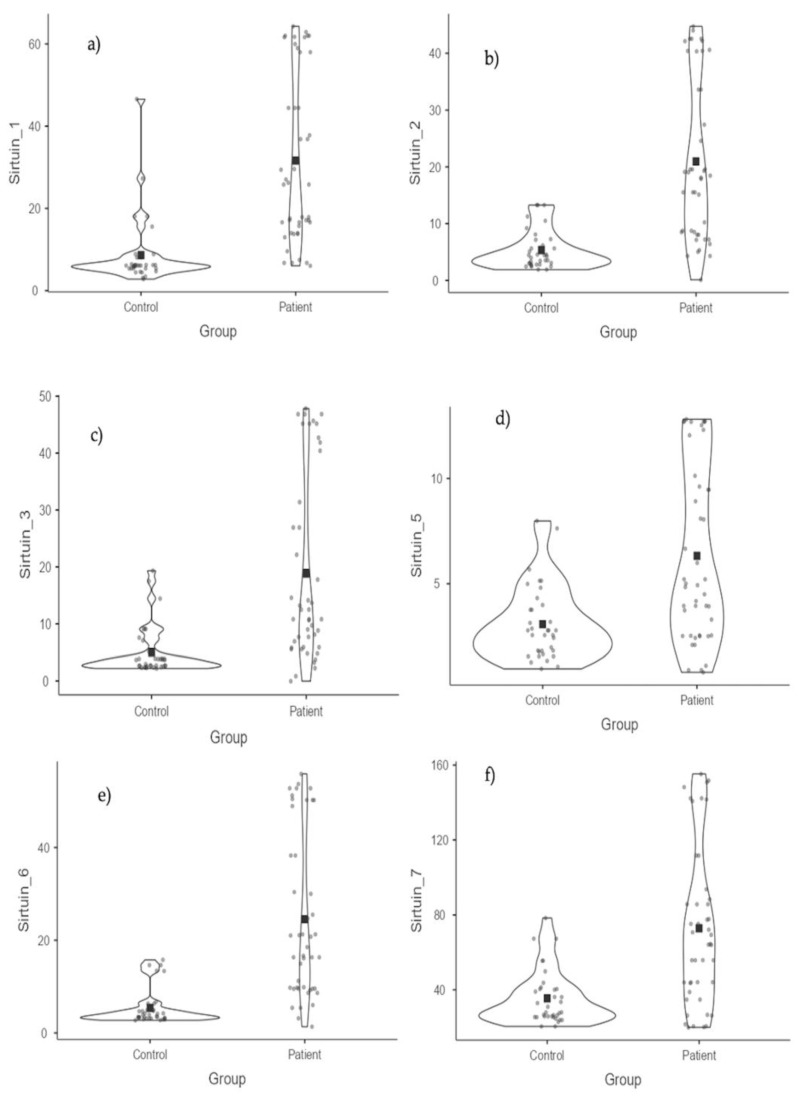
SIRT values according to groups: (**a**) SIRT-1 values; (**b**) SIRT-2 values; (**c**) SIRT-3 values; (**d**) SIRT-5 values; (**e**) SIRT-6 values; and (**f**) SIRT-7 values.

**Figure 2:**
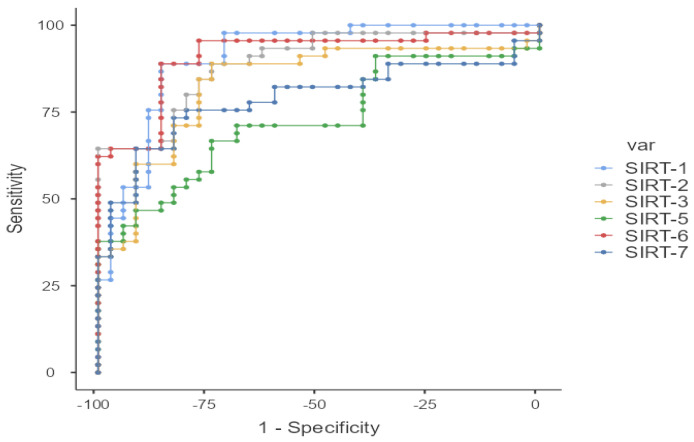
ROC curve for ELISA variables in disease detection.

When comparing the groups, statistically significant differences were found between the patient group and the control group for all SIRT values. The values of SIRT-1, SIRT-2, SIRT-3, SIRT-5, SIRT-6, and SIRT-7 were higher in the patient group compared to the control group. Particularly, significant differences were observed for SIRT-1 (*p* < 0.001), SIRT-2 (*p* < 0.001), SIRT-3 (*p* < 0.001), SIRT-6 (*p* < 0.001), and SIRT-7 (*p* < 0.001), while SIRT-5 also showed a significant difference (*p* = 0.001). These results suggest that SIRT molecules show significant differences in the patient group and could potentially be considered as biomarkers for the disease (Table 2 and Table 3) (Figure 1 and Figure 2).

In early-stage rejection, pre-rejection ELISA values and post-rejection early-stage ELISA values showed statistically significant differences in SIRT-2, SIRT-3, SIRT-5, and SIRT-6 values (*p* < 0.001). No significant difference was found between other molecules (SIRT-1 and SIRT-7). In the late stage, no significant differences were observed in any of the SIRT values (Table 4).

**Figure 3:**
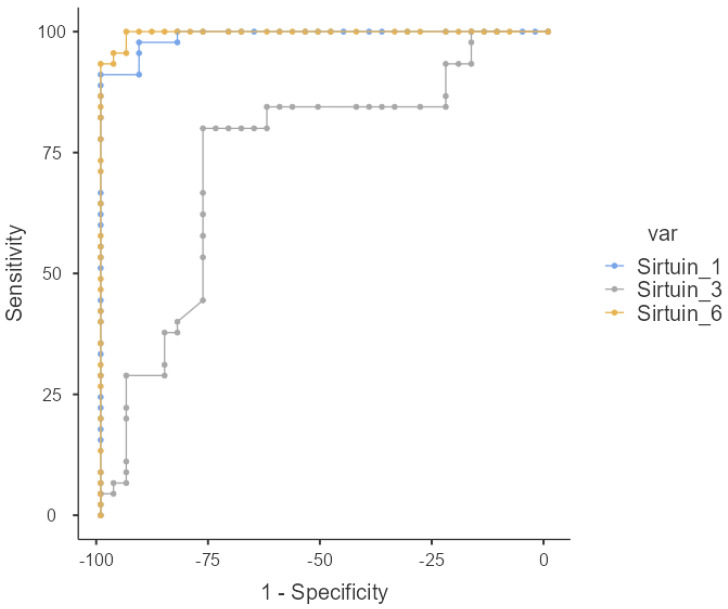
ROC curve for mRNA variables in disease detection.

**Figure 4:**
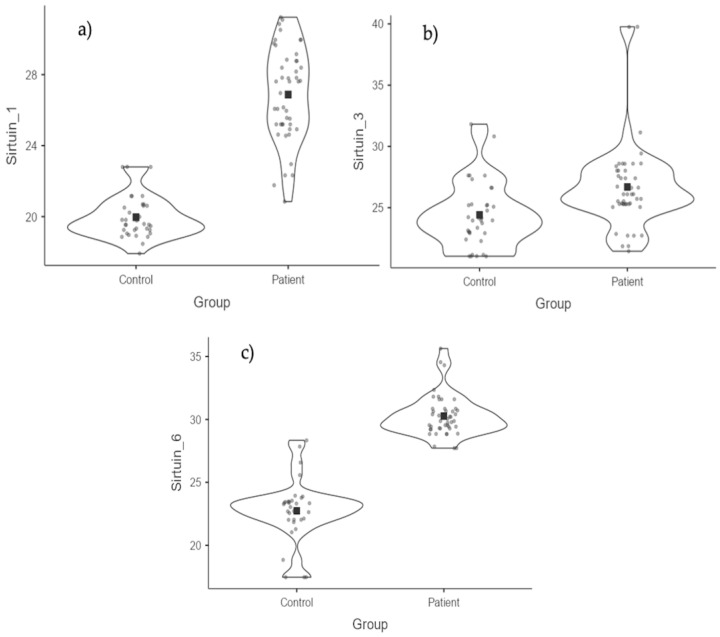
SIRT values according to groups. (**a**) SIRT-1 values. (**b**) SIRT-3 values. (**c**) SIRT-6 values.

In the patient group, the median values of SIRT-1, SIRT-3, and SIRT-6 variables were found to be significantly higher than those of the control group (*p* < 0.001). For SIRT-1, the median value in the patient group was 27.61, and in the control group, it was 19.59; for SIRT-3, the median value in the patient group was 26.1, and in the control group, it was 23.96; for SIRT-6, the median value in the patient group was 29.87, and in the control group, it was 22.72 (Table 5 and Table 6) (Figure 3 and Figure 4).

No statistically significant overall agreement was found between the ELISA and mRNA levels for SIRT-1, SIRT-3, or SIRT-6 in early- or late-stage rejection. However, in the late stage, a statistically significant difference was observed between the median ELISA and mRNA values for SIRT-3 (*p* = 0.021) (Table 7).

Statistically significant differences were found between SIRT-1, SIRT-3, and SIRT-6, but no statistically significant agreement was observed between the median ELISA and mRNA values (*p* > 0.05) (Table 8).

Based on the ELISA results, in the early stage, a statistically significant relationship was found between SIRT-6 and tubulitis (*p* > 0.050). In the late stage, a statistically significant relationship was found between SIRT-3 and interstitial fibrosis (ci) (*p* > 0.050). When no distinction between stages was made, a statistically significant weak positive correlation was found between the SIRT-7 variable and K concentration (r = 0.363; *p* = 0.014) (Table 9).

A positive and moderate significant correlation was found between late-stage mRNA SIRT-3 and interstitial fibrosis (ci) and C4d values (r = 0.432, *p* = 0.024, and r = 0.589, *p* = 0.034, respectively) (Table 10).

## 4. Discussion

In our study, we investigated the role of the SIRT family in relation to acute cellular and humoral rejection during kidney transplantation using ELISA and mRNA methods. We evaluated whether the SIRT family plays a role in acute rejection processes and whether sirtuins could be a potential target for graft outcomes. Our results revealed that SIRT-3, in particular, exhibited significant changes in late-stage rejection processes, suggesting that these changes could serve as potential biomarkers for managing rejection processes. Additionally, the effects of SIRT-3 on C4d expression and interstitial fibrosis were highlighted. These findings indicate that SIRT-3 plays a crucial role in late-stage kidney transplant rejection and may be considered a therapeutic target.

A previous study investigated whether the SIRT family is associated with acute rejection in kidney transplantation and whether changes in the SIRT family can predict the occurrence of acute rejection. In this context, Huali Weng and colleagues utilized transcriptome sequencing results to establish a diagnostic model for acute rejection in kidney transplantation, with these changes being validated through animal experiments. This study was the first to demonstrate the diagnostic value of the SIRT family in acute kidney rejection, suggesting that the findings could provide a new target and theoretical foundation for future research on kidney transplantation. This study stands out as a significant step toward better understanding and managing acute rejection after kidney transplantation [8].

In our study, in terms of demographic characteristics, a statistically significant difference was found in the mean age of the participants in the patient and control groups (*p* = 0.002). The mean age was 42.22 in the patient group and 35.23 in the control group.

Our analysis using ELISA and mRNA methods revealed that SIRT-3, in particular, exhibited significant changes in late-stage acute kidney rejection processes. These findings suggest that the SIRT family may contribute to a better understanding of acute rejection following kidney transplantation and highlight its potential as an important biomarker for future research. Consistent with the study by Huali Weng and colleagues, our results further support the diagnostic value of the SIRT family and its role in managing rejection processes after kidney transplantation.

Weng H et al. first examined the expression landscape of the SIRT family in kidney tissues using the HPA database. Their research revealed that all members of the SIRT family are expressed in kidney tissues. Single-cell sequencing results indicated that the SIRT family could be localized to various cell types, particularly concentrating in renal tubular epithelial cells, suggesting a significant role for the SIRT family in kidney diseases [8].

Xiong W et al. found that UCP1 could mitigate the progression of renal interstitial fibrosis by regulating SIRT3 protein stability and modulating oxidative stress pathways [9]. Similarly, Hong YA et al. provided a detailed summary of the roles and potential mechanisms of SIRT family members in various kidney diseases, emphasizing the SIRT family’s significant potential in the prevention and treatment of kidney diseases [10].

In their study, Weng H et al. conducted a correlation analysis between the SIRT family and 22 different immune cell types, identifying a significant relationship between the SIRT family and immune cell infiltration levels. These findings suggest that the SIRT family may play a crucial role in the acute rejection process of kidney transplantation by regulating immune cells [8].

In our study, it was found that SIRT-3 is particularly associated with interstitial fibrosis and C4d accumulation in the late stage. Additionally, a weak correlation between SIRT-7 and potassium levels was observed. While Weng et al.’s study highlighted the effects of the SIRT family on immune cell infiltration, our study focused more on the potential use of individual members of the SIRT family, specifically in relation to rejection processes, as potential biomarkers.

In contrast to Weng et al.’s work, which addressed the general relationship of the SIRT family with kidney diseases and immune responses, our study is more focused on kidney transplant rejection and emphasizes the potential use of SIRT-3 as a biomarker. In this context, our study offers new insights into the specific role of the SIRT family in kidney transplant rejection and provides a valuable contribution to the literature.

Additionally, Ji L et al. demonstrated that the overexpression of SIRT6 could promote the transformation of M2-type macrophages and alleviate kidney damage associated with diabetic nephropathy [11].

In our study, we specifically focused on the role of SIRT-3 in late-stage acute kidney rejection processes. Through analyses using ELISA and mRNA methods, we examined SIRT-3’s effects on kidney grafts, modulation of immune cells, and relationship with interstitial fibrosis. In this context, we found positive and significant correlations between SIRT-3 and interstitial fibrosis (CI) as well as C4d levels. These findings suggest that the SIRT family, particularly SIRT-3, may play a crucial role in regulating immune responses and rejection processes.

Moreover, similar to the study by Weng H et al., our findings support the idea that the SIRT family could serve as potential biomarkers in immune cells and kidney tissues. Additionally, it is important to emphasize that the findings of Ji L et al. regarding SIRT6 contribute to our study. Both SIRT-3 and SIRT-6 could serve as potential targets in managing kidney diseases, particularly in the rejection processes following kidney transplantation.

Our study examined the mRNA expression levels of the SIRT family and revealed how the expression SIRT family genes change in patients experiencing acute rejection after kidney transplantation. In this context, we found a strong relationship between immune cell infiltration and the SIRT family. While our study focused more on the molecular-level interaction between the SIRT family and immune cells, Weng H et al.’s study adopted a more clinically oriented and modeling-based approach [8].

Overall, the ELISA and mRNA expression levels of SIRT-1, SIRT-2, SIRT-3, SIRT-6, and SIRT-7 were analyzed in rejection patients. AUC calculations were performed for ELISA and mRNA expression, and comparisons between the early and late rejection stages showed that early-stage SIRT-2, SIRT-3, SIRT-5, and SIRT-6 values were significant.

In the patient group, the median values of SIRT-1, SIRT-3, and SIRT-6 were significantly higher than those of the control group (*p* < 0.001). The median value for SIRT-1 was 27.61 in the patient group and 19.59 in the control group; for SIRT-3, it was 26.1 in the patient group and 23.96 in the control group; and for SIRT-6, it was 29.87 in the patient group and 22.72 in the control group.

In early- and late-stage rejection patients, there was no overall statistically significant correlation between the ELISA and mRNA levels of SIRT-1, SIRT-3, and SIRT-6. However, in the late stage, a statistically significant difference was found between the median ELISA and mRNA values for SIRT-3 (*p* = 0.021).

In the examination of the relationship between ELISA variables and other variables in the early and late rejection stages, a statistically significant relationship was observed between SIRT-6 and tubular damage (tubulitis) in the early stage (*p* > 0.050), while in the late stage, a statistically significant relationship was found between SIRT-3 ELISA values and interstitial fibrosis (CI) (*p* > 0.050). When looking at the data without distinguishing between stages, a statistically weak positive correlation was found between the SIRT-7 variable and K values (r = 0.363; *p* = 0.014).

Regarding the relationships between the mRNA expression of SIRT-1, SIRT-3, and SIRT-6 and other variables, significant positive moderate correlations were found between SIRT-3 and interstitial fibrosis (CI) and C4d values (r = 0.432, *p* = 0.024; r = 0.589, *p* = 0.034, respectively).

In our study, a significant increase in SIRT-3 expression and ELISA levels was observed in patients showing late-stage kidney rejection. This increase is particularly associated with C4d positivity and interstitial fibrosis, suggesting that SIRT3 may contribute to tissue damage through the regulation of mitochondrial function and oxidative stress. In the chronic rejection process, pro-inflammatory cytokines secreted by CD4+ T cells and complement activation are known to trigger the fibrotic response. In this context, the increase in SIRT3 may be considered a critical biomarker for the progression of fibrosis and the long-term preservation of graft health.

### Limitations

There are some limitations to our study that provide an important foundation for future research. The evaluation of SIRT-3 as a clinically applicable biomarker is highly feasible, considering current molecular techniques and analytical methods. However, to validate the effectiveness, sensitivity, and specificity of this biomarker, larger-scale, multi-center, and longer-term studies are needed. Such studies have the potential to better elucidate the biological significance and medical applications of SIRT-3 in conditions such as kidney transplant rejection.

First, the lack of data collected before kidney transplantation highlights the need for a more comprehensive examination of early-stage effects. Additionally, the limited sample size of 45 patients suggests that studies conducted with a larger patient group would increase statistical power and strengthen the generalizability of the findings. The limitations of being a single-center study clearly indicate the need for multi-center research to support these results. Investigating the effects of other molecular parameters, especially factors like inflammation and oxidative stress, will contribute to a deeper understanding of the role of the SIRT family in kidney transplantation. Moreover, the short-term follow-up emphasizes the need to determine the long-term effects in future studies.

These limitations can serve as a guide for more comprehensive future studies and strengthen the validity of the findings. Future research may provide more in-depth and broader results based on these initial findings.

## 5. Conclusions

In our study, SIRT1, SIRT2, SIRT3, SIRT5, SIRT6, and SIRT7 levels were examined in kidney transplant patients using ELISA, and SIRT-1, SIRT-3, and SIRT-6 expression was analyzed at the mRNA level. Our findings reveal that the SIRT family exhibits significant changes during the rejection process, with particular emphasis on the role of SIRT-3 in the late stage. These changes suggest that SIRT molecules could serve as potential biomarkers for rejection management. The absence of such studies in the literature highlights the originality and importance of our work, particularly given the limited data on the role of the SIRT family in kidney transplantation. Additionally, the clinical significance of these findings could enable the development of more personalized and effective approaches in treatment and patient management.

## Figures and Tables

**Table 1:** Kidney transplant rejection patients’ demographic and clinical characteristics.

	Patient
Type of Rejection	
Cellular	18 (40)
Humoral	27 (60)
Rejection	
Present	45 (100)
Gender	
Male	31 (68.9)
Female	14 (31.1)
Mode of Transplantation	
Live	26 (57.8)
Cadaver	19 (42.2)
Degree of HLA Matching	
1A	19 (46.3)
1B	25 (61)
1DR	23 (56.1)
2A	6 (14.6)
2B	5 (12.2)
2DR	13 (31.7)
Creatinine (mg/dL)	2.81 (0.89–15.2)
Survival Time	0.8 (−4.42–13.8)
Age	42.22 ± 13.41
Pre-Rejection Creatinine Level (mg/dL)	6.87 ± 3.01
CRP (mg/L)	9.8 (1.9–105.9)
Urea (mg/dL)	101.3 ± 50.44
Na (mmol/L)	137 (114–152.3)
K (mmol/L)	4.25 (3.16–6.33)
BUN (mg/dL)	47.24 ± 23.42

Mean ± standard deviation; median (minimum–maximum). CRP: C-Reactive Protein, Na: Sodium, K: Potassium, BUN: Blood Urea Nitrogen.

**Table 2:** Comparison of ELISA (pre-rejection) variables according to groups.

	Patient (*n* = 45)	Control (*n* = 35)	Test Statistic	*p* *
SIRT-1	25.81 (6.05–64.26)	6.12 (2.82–46.57)	146	<0.001
SIRT-2	18.45 (0.11–44.72)	4.28 (1.91–13.28)	171	<0.001
SIRT-3	10.83 (−0.02–47.83)	3.7 (2.22–19.34)	267	<0.001
SIRT-5	4.93 (0.81–12.8)	2.79 (0.97–7.98)	430	0.001
SIRT-6	18.5 (1.33–55.85)	3.87 (2.7–15.76)	141	<0.001
SIRT-7	64.42 (19.91–155.26)	27.96 (20.44–78.31)	335	<0.001

* Mann–Whitney U-Test; median (minimum–maximum). SIRT-1: Sirtuin 1. SIRT-2: Sirtuin 2. SIRT-3: Sirtuin 3. SIRT-5: Sirtuin 5. SIRT-6: Sirtuin 6. SIRT-7: Sirtuin 7.

**Table 3:** Determination of the cutoff value for ELISA (baseline) variables in identifying the patient group.

	Cutoff Value	AUC (95% CI)	*p*	Sensitivity (%)	Specificity (%)	PPV (%)	NPV (%)	DeLong Test
SIRT-1	≥9.59	0.907 ^b^ (0.84–0.975)	<0.001	88.89%	85.71%	88.89%	85.71%	<0.001
SIRT-2	≥15.12	0.891 ^bd^ (0.821–0.961)	<0.001	64.44%	100%	100%	68.63%	
SIRT-3	≥4.82	0.830 ^cd^ (0.737–0.924)	<0.001	88.89%	74.29%	81.63%	83.87%	
SIRT-5	≥3.93	0.727 ^a^ (0.616–0.837)	0.001	66.67%	74.29%	76.92%	63.41%	
SIRT-6	≥8.62	0.910 ^b^ (0.844–0.977)	<0.001	88.89%	85.71%	88.89%	85.71%	
SIRT-7	≥43.93	0.787 ^ac^ (0.684–0.89)	<0.001	73.33%	82.86%	84.62%	70.73%	

^a–d^: There is no difference between AUC values with the same letter. SIRT-1: Sirtuin 1. SIRT-2: Sirtuin 2. SIRT-3: Sirtuin 3. SIRT-5: Sirtuin 5. SIRT-6: Sirtuin 6. SIRT-7: Sirtuin 7.

**Table 4:** Comparison of ELISA variables between pre-rejection and early/late rejection values.

Period		Pre-Rejection ELISA Values	Post-Rejection Early/Late Phase ELISA Values	Test Statistic	*p* *
Early-Phase Rejection (0–6 month)	SIRT-1	27.83 (9.59–61.98)	49.93 (7.31–62.76)	−1.415	0.157
	SIRT-2	19.43 (5.04–42.55)	33.77 (3.77–43.8)	−2.199	0.028
	SIRT-3	13.93 (3.25–47.83)	33.45 (2.11–48.64)	−2.025	0.043
	SIRT-5	5.11 (1.12–12.72)	11.86 (6.25–16.75)	−2.765	0.006
	SIRT-6	21.04 (1.33–52.76)	40.52 (23.68–53.65)	−2.33	0.020
	SIRT-7	73.67 (19.91–150.84)	128.72 (18.49–153.62)	−1.546	0.122
Late-Phase Rejection (>6 month)	SIRT-1	17.88 (6.05–64.26)	22.47 (6.24–63.04)	−0.553	0.581
	SIRT-2	15.52 (0.11–44.72)	15.03 (0.24–43.38)	−0.192	0.848
	SIRT-3	9.03 (−0.02–46.85)	11.3 (−0.05–48.64)	−0.264	0.792
	SIRT-5	4.18 (0.81–12.8)	4.71 (1.01–12.81)	−0.12	0.904
	SIRT-6	16.3 (3.14–55.85)	18.93 (2.38–54.32)	−0.24	0.810
	SIRT-7	55.79 (20.08–155.26)	63.23 (20.75–155.13)	−0.769	0.442

* Wilcoxon Test; median (minimum–maximum). SIRT-1: Sirtuin 1. SIRT-2: Sirtuin 2. SIRT-3: Sirtuin 3. SIRT-5: Sirtuin 5. SIRT-6: Sirtuin 6. SIRT-7: Sirtuin 7.

**Table 5:** Comparison of mRNA variables according to groups.

	Patient (*n* = 45)	Control (*n* = 35)	Test Statistic	*p*
SIRT-1	27.61 (20.85–31.23)	19.59 (17.92–22.8)	15.0	<0.001 **
SIRT-3	26.1 (21.45–39.76)	23.96 (21.04–31.82)	410.0	<0.001 **
SIRT-6	29.87 (27.72–35.63)	22.72 (17.48–28.34)	5.0	<0.001 **

** Mann–Whitney U-Test; mean ± standard deviation; median (minimum–maximum). SIRT-1: Sirtuin 1. SIRT-3: Sirtuin 3. SIRT-6: Sirtuin 6.

**Table 6:** Determination of the cutoff value for mRNA variables in identifying the patient group.

	Cutoff Value	AUC (95% CI)	*p*	Sensitivity (%)	Specificity (%)	PPV (%)	NPV (%)	DeLong Test
SIRT-1	≥22.96	0.990 ^a^ (0.977–1)	<0.001	91.11%	100%	100%	89.74%	
SIRT-3	≥25.3	0.740 ^b^ (0.625–0.855)	<0.001	80%	77.14%	81.82%	75%	
SIRT-6	≥27.72	0.997 ^a^ (0.991–1)	<0.001	100%	94.29%	95.74%	100%	

^a,b^: There is no difference between AUC values with the same letter. SIRT-1: Sirtuin 1. SIRT-3: Sirtuin 3. SIRT-6: Sirtuin 6.

**Table 7:** Agreement and comparison of early- and late-stage rejection ELISA and mRNA variables in the patient group.

Period		ELISA	mRNA	ICC (95% CI)/*p*	Test Statistic	*p* *
Early-Phase Rejection (0–6 month)	SIRT-1	27.83 (9.59–61.98)	28.11 (22.33–31.09)	0.053 (−0.413–0.496)/0.415	−1.154	0.248
	SIRT-3	13.93 (3.25–47.83)	27.72 (21.86–39.76)	0.173 (−0.307–0.582)/0.240	−1.328	0.184
	SIRT-6	21.04 (1.33–52.76)	29.96 (27.72–34.55)	−0.018 (−0.47–0.441)/0.529	−0.37	0.711
Late-Phase Rejection (>6 month)	SIRT-1	17.88 (6.05–64.26)	26.07 (20.85–31.23)	−0.001 (−0.374–0.373)/0.501	−0.192	0.848
	SIRT-3	9.03 (−0.02–46.85)	25.53 (21.45–39.76)	−0.044 (−0.411–0.336)/0.588	−2.306	0.021
	SIRT-6	16.3 (3.14–55.85)	29.87 (27.72–35.63)	−0.023 (−0.394–0.354)/0.547	−1.826	0.068

* Wilcoxon Test; median (minimum–maximum); ICC: Intraclass Correlation Coefficient. SIRT-1: Sirtuin 1. SIRT-3: Sirtuin 3. SIRT-6: Sirtuin 6.

**Table 8:** Agreement and comparison of ELISA and mRNA variables in the control group.

	ELISA	mRNA	ICC (95% CI)/*p*	Test Statistic	*p* *
SIRT-1	6.12 (2.82–46.57)	19.59 (17.92–22.8)	0.003 (−0.326–0.332)/0.493	−4.521	<0.001
SIRT-3	3.7 (2.22–19.34)	23.96 (21.04–31.82)	−0.26 (−0.543–0.075)/0.937	−5.16	<0.001
SIRT-6	3.87 (2.7–15.76)	22.72 (17.48–28.34)	0.153 (−0.185–0.459)/0.186	−5.159	<0.001

* Wilcoxon Test; median (minimum–maximum); ICC: Intraclass Correlation Coefficient. SIRT-1: Sirtuin 1. SIRT-3: Sirtuin 3. SIRT-6: Sirtuin 6.

**Table 9:** Examination of the relationship between ELISA variables and other variables in early and late rejection stages.

Period			SIRT-1	SIRT-2	SIRT-3	SIRT-5	SIRT-6	SIRT-7
Early-Phase Rejection (0–6 month)	CRP (mg/L)	r	0.393	0.517	0.350	0.067	0.433	0.433
		*p*	0.295	0.154	0.356	0.865	0.244	0.244
	Urea (mg/dL)	r	−0.052	−0.030	−0.191	−0.088	−0.063	−0.030
		*p*	0.839	0.906	0.448	0.729	0.804	0.906
	Na (mmol/L)	r	0.136	0.040	0.108	0.068	0.008	0.114
		*p*	0.589	0.875	0.669	0.790	0.974	0.651
	K (mmol/L)	r	0.174	0.323	0.092	0.148	0.236	0.346
		*p*	0.491	0.191	0.717	0.559	0.345	0.160
	BUN (mg/dL)	r	−0.052	−0.030	−0.191	−0.088	−0.063	−0.030
		*p*	0.839	0.906	0.448	0.729	0.804	0.906
	Interstitial Inflammation (i)	r	−0.136	−0.081	0.041	−0.271	−0.081	−0.122
		*p*	0.659	0.792	0.895	0.371	0.792	0.692
	Tubulitis (t)	r	0.035	0.000	0.035	−0.174	0.170	0.105
		*p*	0.902	1.000	0.902	0.534	**0.039**	0.710
	Glomerulitis (q)	r	0.000	−0.257	−0.077	0.000	−0.129	0.000
		*p*	1.000	0.539	0.856	1.000	0.762	1.000
	Peritubular Capillaritis (ptc)	r	−0.452	−0.486	−0.203	−0.483	−0.343	−0.203
		*p*	0.261	0.222	0.630	0.225	0.406	0.630
	C4d	r	−0.278	−0.679	−0.370	−0.370	−0.525	−0.123
		*p*	0.594	0.138	0.470	0.470	0.285	0.816
	Interstitial Fibrosis (ci)	r	−0.206	−0.206	0.059	−0.206	−0.059	−0.088
		*p*	0.695	0.695	0.912	0.695	0.912	0.868
Late-Phase Rejection (>6 month)	CRP (mg/L)	r	0.049	0.245	0.282	0.303	0.264	0.117
		*p*	0.848	0.327	0.256	0.221	0.290	0.644
	Urea (mg/dL)	r	0.106	0.138	0.209	0.190	0.137	0.204
		*p*	0.597	0.491	0.295	0.343	0.497	0.308
	Na (mmol/L)	r	−0.198	−0.123	0.010	−0.139	−0.075	−0.062
		*p*	0.323	0.541	0.962	0.491	0.709	0.757
	K (mmol/L)	r	0.215	0.146	0.024	0.118	0.175	0.279
		*p*	0.281	0.467	0.907	0.558	0.383	0.159
	BUN (mg/dL)	r	0.111	0.145	0.211	0.203	0.145	0.211
		*p*	0.582	0.472	0.291	0.309	0.470	0.290
	Interstitial Inflammation (i)	r	−0.158	−0.158	−0.356	−0.317	−0.185	−0.211
		*p*	0.544	0.544	0.161	0.215	0.478	0.416
	Tubulitis (t)	r	0.222	0.164	0.202	0.231	0.145	0.183
		*p*	0.362	0.503	0.406	0.341	0.555	0.453
	Glomerulitis (q)	r	−0.348	−0.348	−0.209	−0.400	−0.209	−0.226
		*p*	0.294	0.294	0.538	0.222	0.538	0.503
	Peritubular Capillaritis (ptc)	r	−0.176	−0.308	−0.141	−0.120	−0.345	−0.271
		*p*	0.514	0.245	0.602	0.659	0.190	0.309
	C4d	r	0.278	0.297	0.384	0.269	0.269	0.182
		*p*	0.357	0.324	0.195	0.375	0.375	0.551
	Interstitial Fibrosis (ci)	r	0.134	−0.114	0.203	−0.203	−0.203	−0.025
		*p*	0.752	0.788	**0.021**	0.630	0.630	0.952
Pre-Rejection	CRP (mg/L)	r	0.139	0.306	0.247	0.245	0.308	0.241
		*p*	0.490	0.121	0.214	0.217	0.118	0.227
	Urea (mg/dL)	r	0.129	0.149	0.141	0.159	0.145	0.221
		*p*	0.397	0.327	0.357	0.297	0.343	0.145
	Na (mmol/L)	r	−0.088	−0.072	0.010	−0.099	−0.073	−0.026
		*p*	0.564	0.638	0.948	0.520	0.634	0.865
	K (mmol/L)	r	0.262	0.232	0.135	0.227	0.227	0.363
		*p*	0.082	0.125	0.376	0.134	0.134	**0.014**
	Blood Urea Nitrogen	r	0.130	0.150	0.140	0.163	0.146	0.222
		*p*	0.396	0.325	0.360	0.285	0.337	0.142
	Interstitial Inflammation (i)	r	−0.158	−0.129	−0.198	−0.265	−0.137	−0.196
		*p*	0.405	0.498	0.295	0.156	0.471	0.298
	Tubulitis (t)	r	0.117	0.074	0.130	0.025	0.043	0.123
		*p*	0.509	0.677	0.465	0.890	0.808	0.487
	Glomerulitis (q)	r	−0.086	−0.154	−0.041	−0.186	−0.117	−0.071
		*p*	0.725	0.529	0.866	0.447	0.633	0.772
	Peritubular Capillaritis (ptc)	r	−0.254	−0.312	−0.153	−0.199	−0.327	−0.218
		*p*	0.231	0.138	0.476	0.352	0.118	0.305
	C4d	r	0.000	−0.051	0.098	−0.025	−0.058	0.000
		*p*	0.998	0.834	0.689	0.919	0.813	0.998
	Interstitial Fibrosis (ci)	r	0.038	0.044	0.021	−0.094	−0.032	0.065
		*p*	0.897	0.882	0.944	0.748	0.913	0.827

r: Spearman’s rho Correlation Coefficient, CRP: C-Reactive Protein, Na: Sodium, K: Potassium, BUN: Blood Urea Nitrogen.

**Table 10:** Examination of the relationship between mRNA variables and other variables.

			SIRT-1	SIRT-3	SIRT-6
Early-stage rejection (0–6 month)	CRP (mg/L)	r	0.340	0.500 *	0.182
		*p*	0.371	0.170	0.640
	Urea (mg/dL)	r	0.227	0.234 *	−0.022
		*p*	0.365	0.350	0.930
	Na (mmol/L)	r	0.176 *	−0.281 *	0.093 *
		*p*	0.485	0.258	0.715
	K (mmol/L)	r	−0.072	−0.306 *	−0.245
		*p*	0.777	0.216	0.327
	BUN (mg/dL)	r	0.229	0.234 *	−0.024
		*p*	0.360	0.350	0.924
	Interstitial Inflammation (i)	r	0.027 *	0.476 *	0.176 *
		*p*	0.930	0.100	0.565
	Tubulitis (t)	r	−0.035 *	−0.035 *	−0.087 *
		*p*	0.901	0.901	0.757
	Glomerulitis (q)	r	0.180 *	−0.051 *	0.360 *
		*p*	0.670	0.904	0.381
	Peritubular Capillaritis (ptc)	r	0.327 *	−0.078 *	0.203 *
		*p*	0.429	0.854	0.630
	C4d	r	0.041	−0.463 *	−0.178
		*p*	0.939	0.355	0.736
	Interstitial Fibrosis (ci)	r	−0.038	−0.029 *	0.087
		*p*	0.944	0.956	0.869
Late-stage rejection (>6 month)	CRP (mg/L)	r	0.161 *	0.204 *	0.209 *
		*p*	0.523	0.417	0.406
	Urea (mg/dL)	r	0.022	−0.133 *	−0.053 *
		*p*	0.912	0.508	0.791
	Interstitial Fibrosis (ci)	r	−0.197	0.432 *	−0.261 *
		*p*	0.326	**0.024**	0.188
	K (mmol/L)	r	0.261	0.029 *	0.083 *
		*p*	0.188	0.885	0.681
	BUN (mg/dL)	r	0.020	−0.128 *	−0.048 *
		*p*	0.919	0.525	0.814
	Interstitial Inflammation (i)	r	−0.079 *	−0.345 *	0.238 *
		*p*	0.763	0.175	0.358
	Tubulitis (t)	r	−0.212 *	−0.165 *	−0.154 *
		*p*	0.384	0.500	0.529
	Glomerulitis (q)	r	0.017 *	0.279 *	−0.541 *
		*p*	0.959	0.406	0.086
	Peritubular Capillaritis (ptc)	r	−0.180 *	0.226 *	0.067 *
		*p*	0.505	0.399	0.805
	C4d	r	−0.048 *	0.589 *	−0.288 *
		*p*	0.877	**0.034**	0.340
	Na (mmol/L)	r	0.035	−0.071 *	−0.152 *
		*p*	0.934	0.868	0.719

r: Pearson’s Correlation Coefficient; r *: Spearman’s rho Correlation Coefficient. CRP: C-Reactive Protein, Na: Sodium, K: Potassium, BUN: Blood Urea Nitrogen.

## Data Availability

Data are available from the corresponding author upon reasonable request.
